# Pardon our dust: remodeling care to serve you better

**DOI:** 10.1186/1940-0640-10-S1-A37

**Published:** 2015-02-20

**Authors:** Dennis McCarty, Traci Rieckmann, Stephanie Renfro, Kate Garvey, K John McConnell

**Affiliations:** 1 Health Services Research, Oregon Health & Science University, Portland, OR, 97239, USA

## Overview

The Oregon Health Plan (Medicaid), a national leader in health-care transformation, contracts with 16 regional Coordinated Care Organizations (CCOs) to provide integrated medical, behavioral, and dental care in patient-centered primary care homes. The transformation seeks increased access to primary care, better control of health-care cost increases, and improved health outcomes using global budgets and shared savings to promote quality of care rather than quantity of care.

## Methods

A mixed-methods analysis assesses the implementation of CCOs and the impacts on treatment for alcohol and drug use disorders through qualitative Interviews with stakeholders in each CCO and quantitative analysis of Medicaid encounter data.

## Results

During the first year of implementation, qualitative interviews with the 16 CCOs and participating addiction treatment programs suggest little systematic attention to addiction treatment issues. Baseline data on screening for alcohol and drug use (less than 3% of adult patients have a CPT code for screening) suggest CCOs have not yet incorporated screening and intervention services into clinical practice. There is little change, moreover, in the use of medications to treat alcohol and drug use disorders and in the number of patients treated for alcohol and drug use disorders. A stakeholder observed in a public meeting, “Usually when a clinic remodels, there is a sign asking patients to ‘Pardon our dust: Remodeling to serve you better.’ The Oregon Health Plan is undergoing a major transformation without signs alerting patients about the changes.”

## Conclusions

Oregon’s ambitious agreement with the Federal Government seeks to reduce its rate of spending growth by 2 percentage points without diminishing the quality of care. This arrangement represents one of the most significant efforts to slow health spending and transform the delivery system. Many of the highest cost patients have untreated mental health, alcohol, and drug use disorders. The Oregon health-care transformation offers opportunity to facilitate integrated care for mental health and substance use disorders. Slow implementation and organizational reluctance to change appear to inhibit progress.

